# Epidemic surveillance in a low resource setting: lessons from an evaluation of the Solomon Islands syndromic surveillance system, 2017

**DOI:** 10.1186/s12889-018-6295-7

**Published:** 2018-12-20

**Authors:** Adam T. Craig, Cynthia A. Joshua, Alison R. Sio, Mark Donoghoe, Brigid Betz-Stablein, Nemia Bainivalu, Tenneth Dalipanda, John Kaldor, Alexander E. Rosewell, Gill Schierhout

**Affiliations:** 1 University of New South Wales, Sydney, NSW 2052 Australia; 2 Solomon Islands Ministry of Health and Medical Services, Chinatown, Honiara Solomon Islands

**Keywords:** Surveillance, Evaluation, Communicable disease, Syndromic surveillance, Outbreak, Solomon Islands, Pacific islands, DHIS2, Small island developing states, IHR (2005)

## Abstract

**Background:**

Solomon Islands is one of the least developed countries in the world. Recognising that timely detection of outbreaks is needed to enable early and effective response to disease outbreaks, the Solomon Islands government introduced a simple syndromic surveillance system in 2011. We conducted the first evaluation of the system and the first exploration of a national experience within the broader multi-country Pacific Syndromic Surveillance System to determine if it is meeting its objectives and to identify opportunities for improvement.

**Methods:**

We used a multi-method approach involving retrospective data collection and statistical analysis, modelling, qualitative research and observational methods.

**Results:**

We found that the system was well accepted, highly relied upon and designed to account for contextual limitations. We found the syndromic algorithm used to identify outbreaks was moderately sensitive, detecting 11.8% (IQR: 6.3–25.0%), 21.3% (IQR: 10.3–36.8%), 27.5% (IQR: 12.8–52.3%) and 40.5% (IQR: 13.5–65.7%) of outbreaks that caused small, moderate, large and very large increases in case presentations to health facilities, respectively. The false alert rate was 10.8% (IQR: 4.8–24.5%). Rural coverage of the system was poor. Limited workforce, surveillance resourcing and other ‘upstream’ health system factors constrained performance.

**Conclusions:**

The system has made a significant contribution to public health security in Solomon Islands, but remains insufficiently sensitive to detect small-moderate sized outbreaks and hence should not be relied upon as a stand-alone surveillance strategy. Rather, the system should sit within a complementary suite of early warning surveillance activities including event-based, in-patient- and laboratory-based surveillance methods. Future investments need to find a balance between actions to address the technical and systems issues that constrain performance while maintaining simplicity and hence sustainability.

**Electronic supplementary material:**

The online version of this article (10.1186/s12889-018-6295-7) contains supplementary material, which is available to authorized users.

## Background

Infectious diseases place a significant burden on global health accounting for an estimated 16% of deaths and tens-of-millions of healthy years of life lost, primarily in low-middle income countries [1–3]. Despite this, many developing countries lack the resources, systems and infrastructure required to implement comprehensive multi-faceted early warning surveillance strategies for infectious diseases of outbreak potential; and hence, rely on relatively rudimentary syndromic surveillance methods for their detection [4–6].

**Fig. 1:**
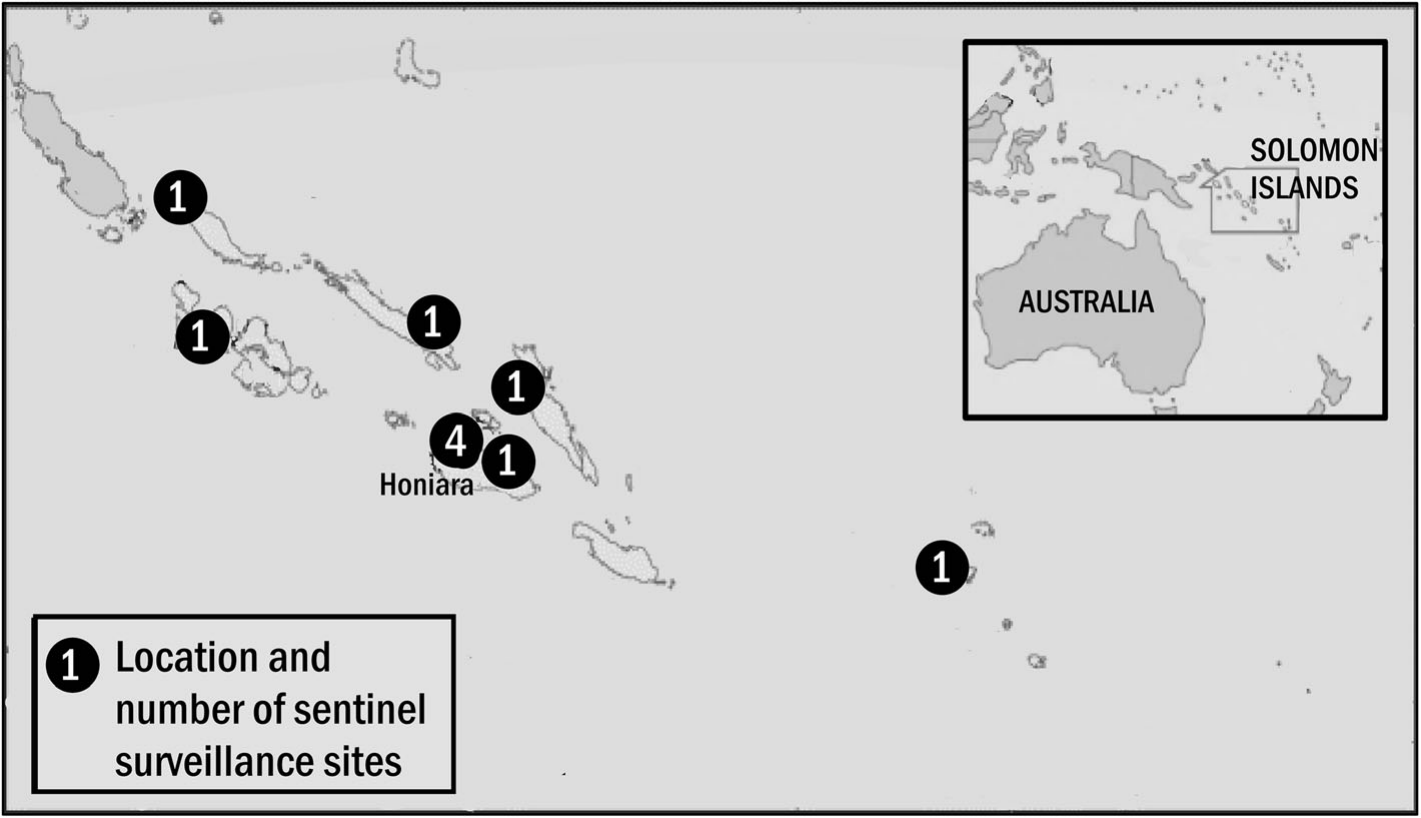
Presents the geographic distribution of sentinel sites that routinely collect data for the Solomon Islands Syndromic Surveillance System. Each site collects data on patient presentations meeting any of five syndrome categories. The total number of patient presentations meeting each syndrome category is reported to the national Ministry of Health and Medical Services on a weekly basis

Solomon Islands (SI) (Fig. 1) is a Pacific island nation of 653,000 people [7] located in the south-west Pacific Ocean, approximately 1800 km north-east of Australia [8, 9]. Most of the population (78%) reside in rural areas and maintain village-based lifestyles [10]. With a per-capita gross national income of just USD1,561 and a United Nations Human Development Index ranking of 156 of 188 nations, SI is one of the least developed countries in the world [9, 11].

In 2011, while recovering from 6 years of civil unrest that resulted in health sector fragility [6] and recognising vulnerability due to the lack of a formalised routine outbreak early warning detection mechanism [12], the Solomon Islands Government (SIG) implemented a simple syndrome-based outbreak detection strategy known as the SI Syndromic Surveillance System (SI-SSS). Syndromic surveillance can be defined as ‘the real-time (or near real-time) collection, analysis, interpretation and dissemination of health-related data to enable the early identification of the impact (or absence of impact) of potential human or veterinary public health threats that require effective public health action’ [13]. The SI-SSS’s design is based on the broader Pacific-wide Pacific SSS (PSSS) [14]. The objectives of the SI-SSS are “to accurately detect outbreaks in the community quickly so that responses can be initiated promptly, and health impacts minimised” and “to support SI’s compliance with IHR (2005) obligations” The SI-SSS is described in Additional file 1.

The national health authority, the Ministry of Health and Medical Services (MHMS), faces complex challenges in delivering health care to the population. Challenges include a fragmented, fragile and under-resourced health sector with multiple and competing health priorities; the need to respond to the pressures of rapid urbanisation while also delivering care to remote communities; high vulnerability to natural disasters; and increasing rates of non-communicable diseases [15–18].

Infectious and parasitic diseases remain marked contributors to the health burden in SI, causing an estimated 8% of all deaths [17] and more than 10% of disability-adjusted life years lost in 2015 [19]. While no log of outbreak events is available, it is understood that epidemics are common with a number of outbreaks challenging the country in recent years, including rubella in 2012/13 [20]; a rotavirus outbreak associated with > 4000 cases in 2014 [21]; a measles outbreak with 4563 cases in 2014 [16]; a Zika virus outbreak in 2015 [22, 23]; a cluster of meningococcal disease in 2015 [24]; and dengue outbreaks in 2013 [25] and again in 2016–17 [26] associated with more than 7000 and 12,000 suspected cases respectively.

In 2017, as part of an effort to strengthen the SI-SSS’s performance the MHMS requested a review of the system be undertaken. In this paper we present the findings of the first evaluation of the SI-SSS and the first exploration of a national experience within the broader PSSS [27]. We aim to determine how well the SI-SSS is performing in terms of its objectives and identify opportunities for improvement.

## Methods

Drawing on seminal guidance produced by the United States Centres for Disease Control and Prevention [28, 29], the World Health Organization (WHO) [30, 31] and the European Centre for Disease Control [32] a mixed-method evaluation research frame was developed. The following system attributes were considered: outbreak detection sensitivity and false alert rate; timeliness; stability; data quality; representativeness; simplicity; acceptability and usefulness. These terms are defined in Additional file 2.

### Qualitative methods and observation

Interviews using semi-structured questionnaires to characterise stakeholders’ perception of system simplicity, acceptability and usefulness were conducted. Respondents were purposefully selected to ensure experiences from a diverse range of roles were captured. Key informants were required to have at least 12-months experience in a data collection and reporting, analysis or management role within the SI-SSS; or to be a user of information generated by the system; or a technical or financial support of it. Key informants were invited to participate by email or telephone. The data collection tool is available at Additional file 3. Informants included nurses responsible for surveillance data collection and reporting; SI-SSS administrators; managers of related MHMS-delivered public health services (i.e., laboratory, health promotion unit staff); and staff of supporting organisations. Drawing on published literature, data collection tools were developed for each group of respondents and administered using an electronic form designed in GoSurvey (www.gosurvey.in/). Interviews were designed to take no longer than an hour. Resulting data were analysed using an inductive approach [33] to identify and characterise emergent themes.

Qualitative information was augmented with data collected through observation of surveillance practices at four sentinel sites and at the national surveillance unit over a 2-week period.

### Data collection and analysis

Surveillance data for the period 1-January-2012 to 30-April-2017 was extracted from the SI-SSS database and analysed using R Statistical software [34] and Microsoft Excel® for timeliness, data quality and stability. Population data was sourced from the SI National Statistics Office [7] and used to characterise system representativeness.

### Modelling

Assessing the ability of a surveillance system to detect outbreaks is complex and requires both an operational definition of ‘outbreak’ and a gold standard of ‘true’ events for comparison [28, 32], neither of which were available. To address this, we used R to model a range of outbreak signals representing various ways outbreaks in the community may affect patient presentations to health facilities. We based our modelling approach on the work of Buckeridge et al. [35] and Mandl et al. [36].

### Baseline data

Baseline data for each site and syndrome category was extracted from the SI-SSS database. We excluded data from two sites and of one syndrome (prolonged fever) as it was insufficient for analysis. To address gaps in data sets’ time-series we applied a data imputation method replacing reporting periods where no data was collected with estimates determined by calculating the mean of the two preceding and two following reporting periods [37]. As we were not able to definitively determine if, and if so when, true outbreaks were reflected in the baseline data, we applied an approach used by Perrin et al. (2005) [38] that uses robust filtering [39, 40] to estimate the median and standard deviation in a moving 52-week window, and trim observations that fell more than three standard deviations above the estimated median at any time. This approach aimed to exclude unrecognised outbreaks while maintaining data authenticity.

### Simulations

To model a wide range of scenarios, and thereby permitting exploration of the effects of controlled variations of signal characteristics, we generated 16 unique case presentation patterns that may result from outbreaks in the community. The signal characteristics varied were: (i) the number of extra case presentations (magnitude), (ii) the time-period over which extra cases presented (duration); and (iii) temporal distribution of extra cases (distribution). We modelled four different magnitudes (a 50, 100, 150 and 200% increase in expected case presentations), and four different distributions: (i) a single peak event with all additional cases presenting in one reporting period; (ii) a single peak with additional presenting over two reporting periods (i.e., 60% of additional cases presenting in the first reporting period and 40% in the second); (iii) a multi-peak event (i.e., 50% of additional cases presenting in the first reporting period, 20% in the second and 30% in the third); (iv) and a prolonged event (i.e., 10% of cases presenting in the first and fourth reporting period and 40% in the second and third). We define a 50% increase in case presentations as a ‘small’ event, a 100% increase as a ‘moderate’ size event, a 150% increase as a ‘large’ event and a 200% increase as a ‘very large’ event.

### Implementing the model

We superimposed the simulated case presentation patterns on to each baseline at a randomly selected point to produce a test dataset. We repeated this process 500 times to produce 500 unique test datasets for each syndrome category and site (i.e., a total of 16,000 unique datasets). We then fit the SI-SSS syndromic algorithm to each and measured its performance. Results were exported as .csv files and analysed using R and Statistical Program for the Social Sciences (SPSS) [41].

### Performance measures

We calculated two performance measures – the proportion of simulated outbreaks detected (i.e., sensitivity) and the false alert rate. Note that we define a false alert rate and not a false positive rate as we could not assume all signals generated by true outbreaks were excluded from baselines. We use the false alert rate as a proxy for specificity.

As data were not normally distributed we calculated the medians and inter-quartile range of continuous data when reporting summary statistics.

## Results

We interviewed 34 key informants including 12 (35%) nurses responsible for data collection, 18 (53%) MHMS staff with system administration/support and response decision-making roles, and four (12%) staff of agencies external to the MHMS that support the SI-SSS. Nurse respondents were drawn from seven of the ten sentinel health facilities that contribute to the SI-SSS; MHMS respondents included four senior executives, four SSS administer-managers, four laboratory staff and six associated public health program managers. Respondents from supporting organisations included two WHO and two bilateral donor agency staff. The median time respondents had been involved in the SI-SSS was 3 years (IQR: 1–3.5).

### Ability to detect outbreaks

Averaged across all baselines, surveillance sites and outbreak signals tested, we found the syndromic algorithm detected 11.8% (IQR: 6.3–25.0%) of outbreaks that resulted in a small increase in case presentations to health facilities; 21.3% (IQR: 10.3–36.8%) of outbreaks that caused a moderate increase; 27.5% (IQR: 12.8–52.3%) of outbreaks that caused a large increase; and 40.5% (IQR: 13.5–65.7%) of outbreaks that caused a very large increase. The false alert rate was at 10.8% (IQR: 4.8–24.5%).

We found the performance of the syndromic algorithm varied across surveillance sites, with the probability of generating a signal inversely proportional to the signal duration and baseline variance and proportionally to signal magnitude.

The syndromic algorithm was best at detecting very large diarrhoeal and influenza-like illness events, syndrome categories that both had relatively large and stable baselines. In these instances, the algorithm was able to detect 62.2% (IQR: 50.2–73.8%) and 41.3% (IQR: 26.2–60.5%) of events, respectively. Conversely, the algorithm’s ability to detect small outbreaks was poor, particularly if the baseline count was small. A more detailed account of the model’s results is presented in Additional file 4.

### Timeliness and stability

We found a high degree of reporting compliance from sentinel sites to MHMS with 95% (IQR: 89–99%) of sites’ weekly reports received, of which 93% (IQR: 89–97%) were received on time (Table 1). Three sites, the National Referral Hospital (NRH) (the longest participating facility), and two rural hospitals (the most recent facilities to join the SI-SSS), had lower reporting rates of 88, and 84% and 70%, respectively. On investigation, reliance on a single nurse who filled multiple roles, absenteeism, high clinical caseloads, and lack of established in-facility procedures for surveillance were identified as key reasons for poor compliance. More broadly, service interruption due to external factors (e.g., facility closure due to structural damage following storms), technical issues (e.g., phone not working) or staffing shortages, and a perception that surveillance tasks were of less importance than clinical roles were found to be common reasons for delay (or failure) to submit data.

**Table 1:** Information about sentinel sites that contribute to the Solomon Islands Syndromic Surveillance System

	National referral hospital	Hospitals						Community health facilities			Total
Location	Honiara city	Province capital	Province capital	Province capital	Province capital	Province capital	Rural town	Honiara city	Honiara city	Honiara city	National
Projected 2017 population (% of total population) ^a^	–	93,953 (14.4%)	156,787 (24%)	34,197 (5.2%)	24,520 (3.8%)	33,139 (5.1%)	139,164 (21.3%)	84,522 (12.9%)			653,248 (100%)
Services provided	In and out patients, laboratory facilities	In and out patients, laboratory facilities	In and out patients, laboratory facilities	In and out patients, laboratory facilities	In and out patients, laboratory facilities	In and out patients, laboratory facilities	In and out patients	Outpatients	Outpatients	Outpatients	
Operating hours/days a week	24/7	24/7	24/7	24/7	24/7	24/7	24/7	8–4/7	8–4/5	8–4/7	
Bed numbers	~ 300	~ 52	~ 150	~ 20	~ 40	~ 43	10	0	0	0	
Doctors	~ 50	~ 3	~ 3	~ 2	~ 1	~ 2	Visiting once or twice per week	0	0	0	
Mean weekly outpatient through-put (SD)	536.2 (156.0)	207.4 (107.4)	–	157.5 (42.8)	222.6 (48.4)	170.2 (27.4)	390.5 (134.9)	596.7 (139.1)	291.4 (103.3)	542.7 (124.8)	418.6 (190.8)
Joined SI-SSS	Jan-12	Jan-12	Feb-12	Feb-12	Mar-13	Jun-16	Apr-14	Jun-12	Jun-12	Jun-12	
Years involved in SI-SSS	5.0	5.0	4.9	4.9	3.9	0.6	2.7	4.6	4.6	4.6	4.1
Timeliness – proportion of surveillance reports made	88%	93%	100%	100%	98%	70%	84%	99%	92%	98%	95%
Timeliness - proportion of submitted reports received on time	91%	87%	100%	100%	88%	90%	72%	97%	95%	97%	93%

^a^ http://www.statistics.gov.sb/statistics/social-statistics/population

Sentinel sites in Honiara (from which the national surveillance coordinator collected SSS data) had consistently high on-time reporting compliance (96% across all Honiara-based sites). While physical collection of data placed significant burden on the coordinator’s time, and likely delayed the production of surveillance reports, it was seen by many as beneficial as it allowed periodic engagement between the national surveillance coordinator and clinic staff and thereby enabled the building of relationships, creation of opportunity for reinforcing surveillance practices, and allowed the incidental sharing of intelligence. One surveillance nurse said “the [MHMS officer’s] visits each week put a face to the system, we like that” while another said, “because the [surveillance coordinator] comes [to extract SI-SSS data] it is one less thing we have to do, this is helpful”. The surveillance coordinator’s facility visits were identified by some nurse respondents as the principal motivating factor for their willing participation in the SI-SSS. “…their [the national surveillance unit] visits demonstrate to us that they value our work here in the clinic,” one nurse said.

We found the MHMS-led data verification, analysis and the weekly SI-SSS report development to be efficient, with, in the 12-months preceding the evaluation, 94% of reports being distributed within the target 48-h timeframe, with the remainder produced within 3-days. Reasons cited for delays were poor/no internet connectivity and reliance on a single officer for the report’s development.

### Data quality

Through observation and interview we identified that all facilities apply a process of retrospective (at the end of the reporting week) review of limited (1–5 word) diagnosis and treatment notes kept in facility-held treatment logbooks to identify (and tally) case-presentations meeting the surveillance definitions. This practice – implemented to maximise efficiency within the facility – has likely compromised the quality of the data collected. We heard of only one attempt to implement data quality control measures through nurse-colleague peer review; however, results of this measures were not available, and the initiative short-lived.

### Representativeness

**Fig. 2:**
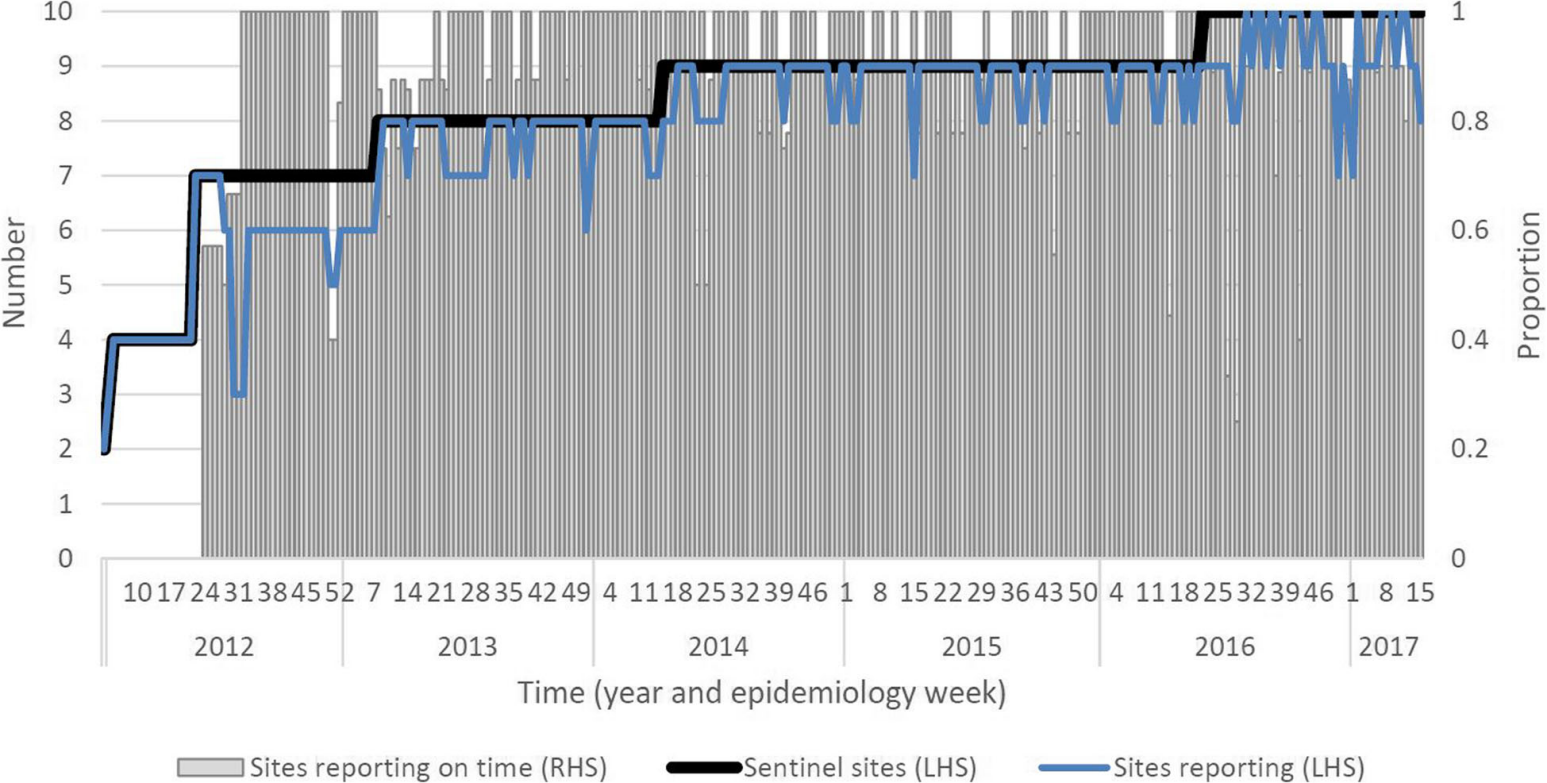
Presents the growth in the number of sentinel surveillance sites participating in the Solomon Islands Syndromic Surveillance System over time (thick solid line), sentinel sites’ reporting compliance (think solid line), and the adherence of sites that make a report to the specified reporting timeframe (columns)

Since its inception in 2011, the SI-SSS has grown from two to ten sentinel sites which, based on reported facility population catchments, is an increase in coverage from 19 to 45% of the population, and an increase from two to seven to 11 administrative jurisdictions (Fig. 2). However, gaps in population representativeness remain in rural areas, where the majority of the SI population reside [10]. The lack of any sentinel sites in Central, Renbel and Makira Provinces (combined population of 87,000,13.3% of SI population) and the limited number of sites on the relatively highly populated islands of Malaita and Guadalcanal are striking limitations.

Figure 1 presents the location of surveillance sites and Table 1 the estimated population catchments, by facility.

### Simplicity

We found the data collection, reporting, analysis and information dissemination components of the system to be straightforward and well understood by officers responsible for the tasks. However, we found the lack of written operational procedures and reliance on a small number of staff (both at the sentinel sites and at MHMS level) is of concern and factors that have the potential to undermine system stability and long-term operational sustainability. We also found the processes that govern public health decision-making in response to surveillance signals to be unclear, an issue that – reportedly –has led to inefficiencies and delays in action when a surveillance signal is generated.

When exploring further, senior MHMS staff reported fragmentation between the surveillance, response and management arms of the health system. Lack of clear administrative responsibility and accountability for action; overlaps and gaps in jurisdictional responsibilities; a reliance on top tiers of government for decision-making; insufficient workforce and resources (especially at the provincial level) to support signal verification, investigations and response efforts; and a hesitancy to fully commit limited health resources without definitive evidence (typically requiring specimens be sent to an overseas laboratory for testing, taking up to 3 weeks) were identified as broader systems factors inhibiting the SI-SSS’s performance.

SI-SSS data collection, collation, analysis and reporting sit outside the National Health Information System (HIS), and some data are collected in duplicate. Reasons given for this included past vertical programming approaches to health sector development inhibiting integration, logistical and technical challenges to integration, perceived delays if SI-SSS data were reliant on the national HIS for data transfer, and inability of the National HIS to quickly upscale in response to major events, if needed.

### Acceptability

Despite the barriers noted above, interviewees from all levels believed that the SI-SSS was of great value to SI generating information adequate for its purpose. One senior MHMS officer commented “…it was due to the groundwork laid by the SSS that we [the MHMS] were able to expand [surveillance activities] during the recent dengue outbreak. Without knowing how to do syndromic surveillance we wouldn’t have been able to identify all the cases we did”. And, “it is during big outbreaks that we find the SSS most valuable,” another manager said, adding “it is during these times we use the data to see trends and locate where things [case numbers] are getting better or worse so we know where to respond”.

More broadly, interviews highlighted that the introduction of the SSS in SI has raised awareness among health workers of the importance of early detection of outbreaks and that the system has “provided a bridge” between the clinical and public health arms of the health sector for outbreak response.

### Supported compliance with the IHR (2005)

We found that the SI-SSS provides the mechanisms required to conduct routine syndromic indicator-based surveillance activities and has demonstrated its capacity to upscale in response to increasing information demands during public health emergencies [21, 25]. We found that the system, while notionally encouraging health workers to report suspected outbreaks, is still in the early stages of systematising event-based surveillance. The capacity of domestic laboratories to confirm aetiologies in suspected disease outbreaks is severely limited, particularly in areas outside Honiara. The MHMS is reliant on basic microscopy and serology methods, rapid diagnostic tests (RDTs) and support from overseas laboratories for confirmatory testing and further analysis.

## Discussion

We report the findings of the first evaluation of the SI-SSS and explore the function of a national experience of the broader PSSS. We found that the SI-SSS provides an accepted, stable and highly valued surveillance mechanism suited to the resource-constrained context of SI, a point highlighted by others as important to maximise implementability and sustainability [5, 6, 27, 42–44]. The SI-SSS is the primary source of information on which MHMS decision makers rely for the detection and monitoring of outbreaks. The system has made a notable contribution to addressing indicator-based surveillance core capacity requirements of the IHR (2005).

Fast and accurate detection of outbreaks are key attributes of a functional outbreak early warning surveillance system. Further, in resource-constrained settings, maintaining a high predictive value positive is important to ensure that the limited resources available for response are used appropriately. The SI-SSS’s outbreak detection mechanism is dependent on the application of a single algorithm which had not previously been validated. We found that the system was moderately successful at detecting large and explosive outbreaks and poor at detecting smaller and protracted events. The implications of these findings for SI (and other states that use similar approaches) is that reliance on an untested syndromic algorithm for the timely detection of small-moderate events may be ill-advised, and development of a broad suite of complementary surveillance activities, including event-, in-patient- and laboratory-based surveillance, is required. Further, we found the approach to be inappropriate for syndrome categories with small baseline counts as variation within these baselines were often larger than the size of the simulated outbreak, leading to a failure of detection. These issues are – in part – a symptom of the small patient throughput of health facilities in SI and therefore needs to be accounted for in the interpretation of information generated by the system. Adoption of a conservative static threshold approach to surveillance signal generation from such data may be more appropriate.

To further explore both the feasibility and performance of statistical signal generation methods we suggest more rigorous analysis that compares a range of syndromic algorithms be undertaken, and that resulting candidate approaches be verified through prospective field testing. Given outbreaks are typically detected locally, analysis should be conducted at the sentinel site level and, where possible, evaluated against data from other systems.

While the geographic and population coverage of the SI-SSS has improved, most rural populations remain outside of the system’s reach, and hence detection of outbreaks in these settings is likely irregular and delayed. Given resource constraints, the ability to expand the number of sites that participate in the SI-SSS is limited and, therefore, concerted effort to reinvigorate a relatively easily scalable and cost-effective EBS strategy is encouraged. This would require revision and promotion of the currently under-utilised national notifiable disease list and associated reporting protocols; establishment of reliable reporting mechanisms; and linkage of EBS with current IBS analysis, signal verification and response procedures.

Syndromic surveillance, as a stand-alone strategy, has significant limitations including a heavy reliance on professional judgement [4–6, 45, 46]. We found domestic laboratory capacity to be insufficient to meet all public health needs, often requiring shipment of specimens to overseas laboratories for definitive testing, causing extended delays and rending resulting information of limited outbreak response decision-making value. A systems development approach to laboratory strengthening including strategies to build national and subnational capacity to test for priority pathogens through expanded use of RDTs and mobile molecular testing equipment (such as mobile polymerase chain reaction (PCR) or geneXpert systems); laboratory twinning; and upskilling surveillance, clinical and laboratory staff on infectious diseases will help address these limitations in SI. Development partners play a central role in supporting these actions.

The frequency at which data are collected and reported (and hence timeliness in which data can be analysed and used) is perhaps the aspect of the system’s design most amenable to improvement. The current protocol of weekly reporting of sites’ data was established in 2011 when the system was new, and principles of simplicity were paramount. Building on recent research that found SI nurses to understand the rationale for more frequent reporting and have a moderate-high willingness to comply with a more frequent reporting regime [47] modification to the frequency of reporting should be considered and trailed. Adoption of mobile technology that enables access to data collected as part of routine patient care (rather than collected solely for surveillance purposes) may be worth further investigation.

The small number of staff available to support SI-SSS is a critical factor limiting system advancement. Opportunities to capitalise on broader workforce investments (such as through in-country training, operational research and mentoring programs, or through participating in field epidemiology training programs) and achieve efficiency through integration of early warning surveillance with broader HIS initiatives should be explored.

To ensure health protection goals are met, it is imperative that measures to improve surveillance performance are balanced with efforts to strengthen response capacity, including addressing governance, human and other up-stream health system ‘roadblocks’. These will require outgoing investment preparedness planning and establishment of clearer event management arrangements that include designation of decision-making responsibilities. Further, establishing standard operating procedures for core surveillance and response activities; building the capacity of national and jurisdictional rapid response teams; and linking outbreak response structures with broader MHMS (and SIG) emergency management arrangements will enhnace the utility of the surveillance system.

The study observed that SI-SSS data collection currently sits outside the national HIS, and some data are being collected in duplicate. The recent introduction of the open-source cloud-based District Health Information System version 2 (https://www.dhis2.org/about/) electronic HIS in SI offers unique opportunities for early warning surveillance to be integrated with, contribute to and draw on existing health informatics.

While we found the existing guidelines for early warning surveillance system evaluation useful as a starting point, we also found limitations. We found a lack of practical guidance for how to adapt the generic advice provided in the guidelines to the operational context of a developing country where measures of success are less quantifiable and where ‘adequate performance’ needs to be framed within what is realistic in the setting. We suggest supplementary guidelines are needed that explicitly address the methodological challenges faced when conducting surveillance system evaluations in complex, small data and limited resource settings.

This study has several limitations. First, the lack of complete historical data on which to base quantitative analysis required we rely on qualitative reports and modelling methods, both of which are prone to bias. Second, as sites’ SSS records are de-identified, the verification of data quality through comparison with medical records was not possible. Third, while we interviewed a broad cross-section of stakeholders, our sample was weighted towards those working in Honiara and hence we may not have comprehensively captured the views of staff from rural areas. Fourth, about one-third of interviews were conducted in group settings where interpersonal dynamics between respondents may have influenced participants’ willingness to contribute. Fifth, due to methodological and data availability limitations we were not able to assess all the attributes of a surveillance system, as outlined in the CDC guidelines [24].

While focused on to SI-SSS, we anticipate that the insights presented in this paper will be of value to practitioners working to strengthen outbreak early warning surveillance systems in other low-resource settings, in particular, in other Small Island Developing States [48].

## Conclusions

In its 6 years of operation the SI-SSS has made a significant contribution to improving public health security in SI. Our evaluation found that the SI-SSS is performing well in certain attributes and highlighted priorities for system enhancement. We found that the SI-SSS provides a suitable mechanism through which the SIG has been able to meet part of their surveillance-related obligations under the IHR (2005). We conclude that syndromic surveillance, while a useful and a feasible surveillance tool suitable for limited resource settings, is insufficiently sensitive to small-moderate size outbreaks and hence should not be relied upon as a stand-alone surveillance strategy. Rather, the syndromic surveillance should sit within a complementary suite of early warning surveillance activities that include event-, in-patient- and laboratory-based surveillance methods.

Looking ahead, future investments need to find a balance between actions to address the technical and systems issues identified in this paper while maintaining simplicity. Where possible, investments should adopt a health system strengthening approach integrating with and capitalising on broader advances in HIS.

## Additional files

Additional file 1: Overview of the Solomon Islands Sydnromic Surveillance System. This file provided a description of the surveillance system under review including the mechanisms buy with data is collected, transferred, and analysed, and how surveillance information is distributed. (PDF 55 kb)

Additional file 2: Definitions of system attributes. This file provided definitions for each system component evaluated and reported in the manuscript. (PDF 50 kb)

Additional file 3: SI-SSS Evaluation data collection tool. This file provided the key informant interview data collection tool used during the evaluation. (PDF 133 kb)

Additional file 4: Results of the performance analysis of the outbreak detection algorithm used in Solomon Islands. This file provides detailed results of the model-based performance evaluation of the outbreak detection algorithm used in Solomon Islands. (PDF 31 kb)

## Abbreviations

EBS: Event-based surveillance
Fig: Figure
HIS: Health Information Systems
IBS: Indicator-based surveillance
IHR: International Health Regulations
IQR: Inter-quartile range
MHMS: Ministry of Health and Medical Services
MS: Microsoft
NRH: National Referral Hospital
PCR: Polymerase chain reaction
PNG: Papua New Guinea
PSSS: Pacific Syndromic Surveillance System
RDT: Rapid diagnostic test
SI: Solomon Islands
SIG: Solomon Islands Government
SI-SSS: Solomon Islands Syndromic Surveillance System
SSS: Syndromic surveillance system
USD: United States dollars
WHO: World Health Organization
