# Supplementary material for: Epidemic surveillance in a low resource setting: lessons from an evaluation of the Solomon Islands syndromic surveillance system, 2017
Source: BMC Public Health. 2018 Dec 20;18:1395. doi: 10.1186/s12889-018-6295-7 (PMC6302379; doi:10.1186/s12889-018-6295-7)
Supplement: Supplementary file 1 — Overview of the Solomon Islands Sydnromic Surveillance System. This file provided a description of the surveillance system under review including the mechanisms buy with data is collected, transferred, and analysed, and how surveillance information is distributed. (PDF 55 kb) [file 12889_2018_6295_MOESM1_ESM.pdf]

Overview of the Solomon Islands Syndromic Surveillance System

The Solomon Islands Syndromic Surveillance System (SI-SSS) involves the collection of pre-diagnosis syndrome-based data from [at the time the analysis was conducted] ten sentinel health facilities including three community clinics; one hospital in the capital city, Honiara; five hospitals in Provincial capitals; and one hospital in a rural setting. Site nurses screen presenting patients for five syndromes (acute fever and rash, dengue-like illness, acute diarrhoea, influenza-like illness, and prolonged fever) and report collected count data to the national health authority, the Ministry of Health and Medical Services, on a weekly basis. Data are analysed using a single algorithm to detect statistical abnormalities that signal potential public health events require verification. The algorithm used is conservative being "a weekly count greater than 90% of all past weekly counts for a given syndrome". Analysis is performed on site and aggregated national data. Signals are verified, and suspected outbreaks brought to the attention of senior health staff who – depending on the information at hand, perceived severity and resource availability – initiate investigation, risk assessment and response measures.

The SI-SSS coordinator produces a weekly report that is distributed to approximately 120 recipients. The report presents data trends highlighting where surveillance signals have been generated. The report also provides a synopsis of ongoing outbreaks and outbreak risks in Solomon Islands, the Pacific region and – if relevant –

the global level. The report is used to disseminate clinical and public health response instructions to health facility staff, if required.

Each week, aggregated national data is sent to the World Health Organization (WHO) Division of Pacific Technical Support where Pacific regional analysis is conducted. WHO produces a weekly Pacific surveillance report that is distributing to >700 Pacific, broader regional and global public health partners via the Pacific Public Health Surveillance Network's Listserv, 'PacNet' (http://goo.gl/03cctM). Data are used for regional and global disease monitoring.
