# Supplementary material for: Epidemic surveillance in a low resource setting: lessons from an evaluation of the Solomon Islands syndromic surveillance system, 2017
Source: BMC Public Health. 2018 Dec 20;18:1395. doi: 10.1186/s12889-018-6295-7 (PMC6302379; doi:10.1186/s12889-018-6295-7)
Supplement: Supplementary file 2 — Definitions of system attributes. This file provided definitions for each system component evaluated and reported in the manuscript. (PDF 50 kb) [file 12889_2018_6295_MOESM2_ESM.pdf]

Definitions of system attributes

Sensitivity -- refers to the proportion of cases of a disease (or other health-related event) detected by the surveillance system…[or]… the ability to detect outbreaks, including the ability to monitor changes in the number of cases over time.

False alarm rate -- refers to the proportion of false positive signals generated by a surveillance system.

Timeliness -- refers to the time taken to transmit information between each step of the surveillance system.

Data quality -- refers to the accuracy of data that is inputted into a system. Adequate data quality is a prerequisite for validity.

Representativeness -- refers to the population and geographic capture of a surveillance system, and a surveillance system's ability to accurately describe the occurrence of events over time and their distribution in the population by place and person.

Simplicity -- refers to both its structure and ease of operation [of a surveillance system].

Acceptability -- refers to the willingness of persons in the sponsoring agency that operates the system and persons outside the sponsoring agency (e.g., persons who are asked to report data) to use the system.

Usefulness -- refers to the contribution to the prevention and control of adverse health-related events, including an improved understanding of the public health implications of such events. In addition, data from a surveillance system can be useful in contributing to performance measures used in needs assessments and accountability systems.

Ref: US CDC Guidelines Working Group (2001): Updated Guidelines for Evaluating Public Health Surveillance Systems. Recommendations from the Guidelines Working Group. In *Morbidity & Mortality Weekly Report* 50 (RR 13), pp. 1–35.
