# Supplementary material for: Epidemic surveillance in a low resource setting: lessons from an evaluation of the Solomon Islands syndromic surveillance system, 2017
Source: BMC Public Health. 2018 Dec 20;18:1395. doi: 10.1186/s12889-018-6295-7 (PMC6302379; doi:10.1186/s12889-018-6295-7)
Supplement: Supplementary file 3 — SI-SSS Evaluation data collection tool. This file provided the key informant interview data collection tool used during the evaluation. (PDF 133 kb) [file 12889_2018_6295_MOESM3_ESM.pdf]

SI-SSS Evaluation - MHMS staff key informant interview data collection tool

a) Administrative data

1. **Date**

   ____

   Example: 15 December 2012

2. **Location/facility name**

   ____

3. **Respondent**

   ____

4. **Informed consent received**

   Mark only one oval.

   - ( ) Yes
   - ( ) No

5. **Informed consent reference number**

   ____

6. **Permission to record the interview?**

   If no, conduct interview and record data in detailed notes

   Mark only one oval.

   - ( ) Yes
   - ( ) No

b) Interview questions

7. **What is your primary role in the Solomon Islands health system?**

   Mark only one oval.

   - ( ) Clinical nurse
   - ( ) Clinical doctor
   - ( ) SI-SSS manger / administrator
   - ( ) MHMS manager
   - ( ) No role within the SI health system but a stakeholder in its function
   - ( ) Other: _______________

8. **What role do you play in the SI-SSS?**
Mark only one oval.

- Collect and report data
- Conduct analysis and produce information
- Manager the day-to-day operation of the system
- Receive and use information generated by the system
- Provide technical and/or financial supporter
- Interested party on the periphery of the SI-SSS but do not play a role in it
- Other: ____________________

9. **How long have you been involved in the SI-SSS?**
Mark only one oval.

- <1 yr
- 1-3 yrs
- >3 yrs

10. **What do you see as the key strengths of the SI-SSS?**

____

11. **What are the SI-SSS's weaknesses?**

____

12. **What do you understand to be the purpose (aims or objectives) of the SI-SSS?**

____

13. **In your view, how well is the SI-SSS meeting this/these purpose/s?**
Mark only one oval.

	1	2	3	4	5	
Not well at all	⬭	⬭	⬭	⬭	⬭	Very well

14. **What factors do you see affecting, either positively or negatively, the SI-SSS's ability to meet this/these purpose/s?**

____

15. **Thinking back, what events, activities or initiatives have lead to a noticeable improvement in the SI-SSS's function?**

____

16. **[For surveillance nurses only] Talk me through the process of collecting and reporting surveillance data.**

____

17. **[For surveillance nurses only] Are there aspects of this process that could be streamlined or simplified? What would be required for this to happen?**

18. **[For surveillance nurses only] Are there written procedures available to guide the data collection and reporting process? If so, how useful are they?**

19. **[For surveillance nurses only] Please provide comment on the usability of the surveillance case definitions, that is, how easy they are to understand and apply? Do you have any difficulties using them?**

20. **[For system managers only] How well integrated is the SI-SSS with other health data collection and information systems in Solomon Islands?**

21. **[For system managers only] Do you feel the case definitions used in the SI-SSS are appropriate (ie, easily understood and applied, sensitive enough, appropriate for the disease profile)?**

22. **[For system managers only] The SI-SSS has an event-based surveillance component. How is event-based surveillance implemented in Solomon Islands? How would you describe its functionality?**

23. **Can you provide examples of how the SI-SSS is contributing to health protection in Solomon Islands?**

24. **Which of the following statements aligns best with your views?**
Mark only one oval.

- ( ) The SI-SSS is able to detect the vast majority of outbreaks, big and small.
- ( ) The SI-SSS is good at picking up big outbreaks but not so good at picking up small ones.
- ( ) It is unclear to me if the SI-SSS is able to pick up outbreak.
- ( ) None of these statements reflect my view. My view is better expressed as... [record in 'other']
- ( ) Other: ______________________

25. **Thinking about the timeliness of data flow through the SI-SSS, are there points in the system where unacceptable delays occur? If so, at what stages?**
Tick all that apply.

- [ ] Data collection
- [ ] Reporting from surveillance sites to MHMS
- [ ] Data analysis and information generation
- [ ] Notifying management of surveillance signals / concern
- [ ] Responding to signals
- [ ] Other: ______________________

26. **What are the causes of these delays and what could be done to address them?**

____

27. **[For system managers only] Talk me through what happens when a surveillance signal is generated?**

____

28. **[For system managers only] Typically, what works well when responding to a surveillance signal? And what doesn't?**

____

29. **[For system managers only] How confident are you in the accuracy of SI-SSS-generated signals?**

____

30. **[For system managers only] What contribution do other source of health data (e.g., laboratory reporting; hospital-based reporting) make to outbreak detection in Solomon Islands?**

____

c) Conclusion

31. **In general, how satisfied are you with the SI-SSS's performance?**
Mark only one oval.

	1	2	3	4	5	
Not very	◯	◯	◯	◯	◯	Very

32. **Is there anything else that you would like to add?**

____

33. **Would you like to receive a copy of the results of this research?**
Mark only one oval.

◯ Yes
◯ No
