# Supplementary material for: Epidemic surveillance in a low resource setting: lessons from an evaluation of the Solomon Islands syndromic surveillance system, 2017
Source: BMC Public Health. 2018 Dec 20;18:1395. doi: 10.1186/s12889-018-6295-7 (PMC6302379; doi:10.1186/s12889-018-6295-7)
Supplement: Supplementary file 4 — Results of the performance analysis of the outbreak detection algorithm used in Solomon Islands. This file provides detailed results of the model-based performance evaluation of the outbreak detection algorithm used in Solomon Islands. (PDF 31 kb) [file 12889_2018_6295_MOESM4_ESM.pdf]

Parameter	Syndrome	Median	Inter-quartile range	
			Lower	Upper
**Magnitude of additional case presentations**				
Small	Acute fever and rash	6.50%	3.50%	17.84%
	Dengue-like illness	15.00%	9.84%	42.84%
	Diarrhoea	21.83%	13.00%	27.50%
	Influenza-like illness	7.00%	3.84%	11.50%
Moderate	Acute fever and rash	7.67%	2.83%	19.50%
	Dengue-like illness	20.84%	11.83%	54.50%
	Diarrhoea	36.50%	31.00%	49.00%
	Influenza-like illness	15.50%	8.67%	30.34%
Large	Acute fever and rash	9.67%	4.34%	19.50%
	Dengue-like illness	21.84%	14.67%	58.17%
	Diarrhoea	49.67%	41.33%	63.50%
	Influenza-like illness	28.17%	17.67%	49.50%
Very large	Acute fever and rash	11.00%	4.83%	20.33%
	Dengue-like illness	22.84%	14.67%	61.33%
	Diarrhoea	62.17%	50.17%	73.84%
	Influenza-like illness	41.34%	26.17%	60.50%
**Duration and temporal distribution of additional cases presentations**				
Single peak event with all cases presenting in 1 reporting period	Acute fever and rash	15.67%	3.67%	26.67%
	Dengue-like illness	24.33%	15.83%	52.50%
	Diarrhoea	35.34%	20.50%	46.67%
	Influenza-like illness	12.17%	6.67%	25.84%
Single peak event with additional cases presenting over 2 reporting periods	Acute fever and rash	11.17%	3.50%	22.34%
	Dengue-like illness	19.17%	13.00%	46.33%
	Diarrhoea	30.50%	19.67%	38.67%
	Influenza-like illness	10.67%	6.33%	21.00%
Multi-peak event with additional cases presenting over 3 reporting periods	Acute fever and rash	12.00%	4.17%	24.00%
	Dengue-like illness	19.67%	11.84%	47.34%
	Diarrhoea	28.00%	19.33%	37.83%
	Influenza-like illness	8.17%	4.00%	20.17%
Prolonged event over 4 reporting periods	Acute fever and rash	9.33%	3.00%	19.67%
	Dengue-like illness	17.33%	12.17%	44.00%
	Diarrhoea	27.17%	18.50%	35.50%
	Influenza-like illness	7.50%	4.00%	18.84%
**False alert rate**				
	Acute fever and rash	4.50%	1.50%	9.34%
	Dengue-like illness	14.50%	8.84%	22.00%
	Diarrhoea	27.00%	20.33%	30.67%
	Influenza-like illness	8.50%	4.34%	15.00%
